# MicroRNA-21, T helper lineage and autoimmunity

**DOI:** 10.18632/oncotarget.3928

**Published:** 2015-04-24

**Authors:** Gopal Murugaiyan, Lucien P. Garo, Howard L. Weiner

**Affiliations:** Ann Romney Center for Neurologic Diseases, Brigham and Women's Hospital and Harvard Medical School, Boston, MA, USA

In response to appropriate stimuli in their microenvironment, naïve CD4^+^ T cells differentiate into one of several T helper cell lineages, such as Th1, Th2, Th17, and Treg cells, as defined by their transcription factor expression, cytokine secretion, and effector functions. Although T helper subsets are known to be regulated by specific transcription factors and cytokines, the molecular basis of T helper cell differentiation, especially the role of microRNA (miRNA) pathways, is incompletely understood. MicroRNAs are short non-coding RNAs that post-transcriptionally modulate the expression of multiple target genes and are implicated in a wide array of cellular and developmental processes, including immune system development.

MicroRNA-21 (miR-21) was one of the earliest identified cancer-promoting “oncomirs,” and therefore much of the research involving this miRNA has focused on its role in tumor promotion [1]; however, recent data also suggest a crucial role for miR-21 in immune system function. MiR-21 is expressed at low levels in T cells and antigen-presenting cells (APCs), but is upregulated in these cells following activation. Although miR-21 seems to have some anti-inflammatory properties at the APC level [2], emerging studies indicate that miR-21 promotes tissue inflammation and autoimmunity by altering the balance of T helper differentiation and function.

**Figure 1:**
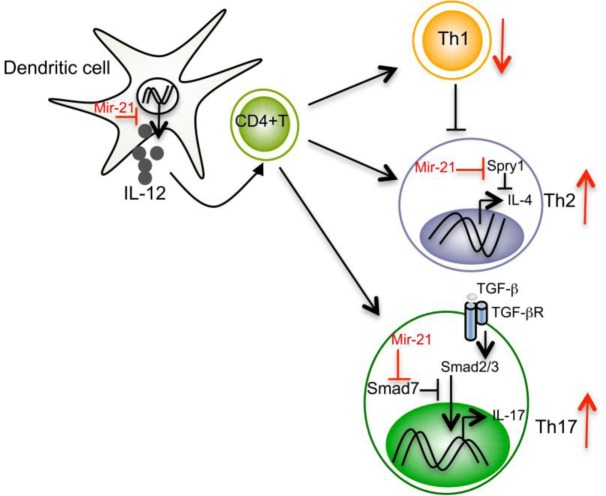
Mir-21 regulation of T helper subset differentiation

Proinflammatory Th1 and anti-inflammatory Th2 cells exist in a balanced state by counter-regulating each other's differentiation and function. MiR-21 has been shown to regulate this balance via multiple pathways [3]. For example, miR-21 is induced in activated DCs and directly targets the mRNA that encodes the p35 subunit of Th1-promoting IL-12, causing miR-21 deficient (miR-21^−/−^) mice to present with increased DC-secreted IL-12 and enhanced Th1 development. Because IL-12 is highly potent at inducing Th1 differentiation and inhibiting Th2 cells, IL-12 inhibition may represent a major mechanism by which miR-21 affects the Th1/Th2 balance. Moreover, by inhibiting Th1 differentiation, miR-21 might indirectly relieve the suppressive effect of Th1-secreted IFN-γ on Th2 cells, further promoting Th2 development (Figure 1). In addition to DC-intrinsic miR-21, T-cell intrinsic miR-21 has been shown to promote Th2 differentiation by inhibiting expression of the Spry1 transcript, a MAP kinase pathway inhibitor. Mice with a deletion of miR-21 show defects in Th2 development and resistance to Th2 mediated allergic airway inflammation. (In contrast to Th2-mediated immediate hypersensitive responses, miR-21 deficiency enhances Th1 development and more delayed type hypersensitivity responses *in vivo*.) In fact, miR-21 has been shown to be one of the most highly upregulated miRNAs in multiple models of experimental asthma and human eosinophilic esophagitis [4]. These data suggest that miR-21 may modulate asthma pathogenesis by shifting T helper differentiation towards the Th2 subtype. However, since the IL-12-Th1 cell response is crucial for protective immunity to various intracellular pathogens, miR-21-mediated shifts in Th1/Th2 balance may also have profound effects on host susceptibility to infection and disease course. To our knowledge, this possibility has yet to be formally investigated.

In addition to regulating Th1 and Th2 cells, miR-21 also affects Th17 differentiation. Most recently, we found that miR-21 expression is specifically elevated in Th17 cells and that T cell-intrinsic expression of miR-21 is important for effective Th17 differentiation [5]. MiR-21 promotes Th17 differentiation by targeting Smad-7, a negative regulator of TGF-β signaling. MiR-21^−/−^ mice show a defect in Th17 differentiation and are resistant to Th17-mediated experimental autoimmune encephalomyelitis (EAE), an animal model of multiple sclerosis (MS). In support of the EAE model, increased expression of miR-21 has been observed in peripheral blood mononuclear cells from MS patients. Although Th1 and Th17 cells are considered to be key participants in EAE and MS, the Th1-signature cytokine IFN-γ and Th17-associated cytokines such as IL-17A, IL-17F, IL-21, and IL-22 have all been shown to be dispensable for the development of EAE. Rather, it is granulocyte macrophage colony-stimulating factor (GM-CSF) which is crucial for the pathogenic functions of Th1 and Th17 cells. Autoreactive Th1 and Th17 cells specifically lacking GM-CSF do not transfer EAE. Interestingly, the almost complete EAE resistance we observed in miR-21^−/−^ mice is associated with reduced GM-CSF in both Th1 and Th17 cells. Consistent with our study, others have found that overexpression of miR-21 *in vivo* leads to elevated levels of multiple proinflammatory cytokines, including IL-17 [6], and that *in vivo* silencing of miR-21 is associated with a reduction in Th17 cells and the transcription factor RAR-related orphan receptor gamma (RORγt) during myocarditis [7].

These and other miR-21-mediated mechanisms seem to affect fundamental immune system functions and are likely involved in a number of other inflammatory diseases (reviewed in [8]). For example, miR-21 has been found to be overexpressed in CD4^+^ T cells derived both from patients with lupus and from lupus-prone MRL/lpr mice, while silencing miR-21 *in vivo* has been shown to ameliorate splenomegaly in lupus mice. Increased expression of miR-21 has also been observed in patients with ulcerative colitis, and most recently, miR-21^−/−^ mice have been shown to be resistant to dextran sulfate sodium (DSS)-induced colitis. Elevated miR-21 levels in T cells from patients with psoriasis have even been shown to correlate with disease activity. In addition, miR-21 expression has been reported in other diseases fueled by chronic inflammation, including atherosclerosis, type 2 diabetes, and cancer.

Together, these studies highlight a stronger role for miR-21 in the pathogenesis of multiple autoimmune and chronic inflammatory disorders. However, the complete molecular mechanisms by which miR-21 regulates these pathologies remains to be investigated. Thus, a better understanding of the role of miR-21 in the innate and adaptive immune systems, during healthy states as well as during infection, chronic inflammation, and autoimmunity, is required before we design any therapeutic strategies aimed at targeting miR-21.
